# Unparalleled mitochondrial heteroplasmy and *Wolbachia* co-infection in the non-model bee, *Amphylaeus morosus*

**DOI:** 10.1016/j.cris.2022.100036

**Published:** 2022-04-20

**Authors:** Olivia K. Davies, James B. Dorey, Mark I. Stevens, Michael G. Gardner, Tessa M. Bradford, Michael P. Schwarz

**Affiliations:** a College of Science and Engineering, Flinders University, SA 5001, Adelaide, Australia; b Biological and Earth Sciences, South Australian Museum, SA 5000, Australia; c Ecology and Evolutionary Biology, Yale University, CT 06511, New Haven, USA; d Center for Biodiversity and Global Change, Yale University, CT 06511, New Haven, USA; e School of Biological Sciences, University of Adelaide, SA 5005, Australia

**Keywords:** Hymenoptera, maternal inheritance, endosymbiont, Hylaeinae, mitochondrial heterogeneity

## Abstract

Mitochondrial heteroplasmy is the occurrence of more than one type of mitochondrial DNA within a single individual. Although generally reported to occur in a small subset of individuals within a species, there are some instances of widespread heteroplasmy across entire populations. *Amphylaeus morosus* is an Australian native bee species in the diverse and cosmopolitan bee family Colletidae. This species has an extensive geographical range along the eastern Australian coast, from southern Queensland to western Victoria, covering approximately 2,000 km. Seventy individuals were collected from five localities across this geographical range and sequenced using Sanger sequencing for the mitochondrial cytochrome c oxidase subunit I (COI) gene. These data indicate that every individual had the same consistent heteroplasmic sites but no other nucleotide variation, suggesting two conserved and widespread heteroplasmic mitogenomes. Ion Torrent shotgun sequencing revealed that heteroplasmy occurred across multiple mitochondrial protein-coding genes and is unlikely explained by transposition of mitochondrial genes into the nuclear genome (NUMTs). DNA sequence data also demonstrated a consistent co-infection of *Wolbachia* across the *A. morosus* distribution with every individual infected with both bacterial strains. Our data are consistent with the presence of two mitogenomes within all individuals examined in this species and suggest a major divergence from standard patterns of mitochondrial inheritance. Because the host's mitogenome and the *Wolbachia* genome are genetically linked through maternal inheritance, we propose three possible hypotheses that could explain maintenance of the widespread and conserved co-occurring bacterial and mitochondrial genomes in this species.

## Introduction

Mitochondrial heteroplasmy is the presence of more than one type of mitochondrial DNA (mtDNA) within a single cell or individual. It is sporadically reported in the literature and can be caused by biparental inheritance of mitogenomes (Gyllensten et al., 1991; Kvist et al., 2003), replication errors (Hoelzel et al., 1993; Irwin et al., 2009), mutagenic processes (Muntaabski et al., 2020), or recombination (Zsurka et al., 2005). Mitochondrial heteroplasmy has generally been viewed as an intermittent, transient condition in natural populations (Song et al., 2008; Hoarau et al., 2002) that might be facilitated by hybridization (e.g. Gandolfi et al. (2017); Mastrantonio et al. (2019)). Mitochondrial heteroplasmy has also been occasionally reported to be widespread in some taxon groups (e.g. chewing lice (Pietan et al. 2016), ticks (Xiong et al. 2013), and isopods (Doublet et al. 2012)). Reports of non-transient forms of heteroplasmy pose problems for our understanding of standard mitochondrial inheritance models.

A common form of mitochondrial heteroplasmy in arthropods is site or point heteroplasmy (Barr et al., 2005), where variation presents as substitutions at single nucleotides, usually occurring at the third codon position (Robison et al., 2015; Nunes et al., 2013). Site heteroplasmy can be maintained within a single heteroplasmic codon (e.g. Doublet et al. (2008); Doublet et al. (2012)) or occur throughout the mitogenome (e.g. Xiong et al. (2013); Meza-Lázaro et al. (2018); Jebb et al. (2018)). In humans, somatic heteroplasmic mutations can become more prevalent with age, often without expressing any clinical symptoms of mitochondrial disease (Ye et al., 2014; Kang et al., 2016). It is likely that other organisms also develop similarly and have varying rates of susceptibility to mitochondrial mutations during their development, through exposure to oxidative damage (Santos et al., 2013; Shokolenko et al., 2014), insufficient repair mechanisms (Kmiec et al., 2006), or errors caused by mitochondrial polymerases during mtDNA replication (Kennedy et al., 2013; Trifunovic et al., 2004). However, these mutations are not necessarily heritable (Ju et al., 2014; Pinto and Moraes, 2015). Extensive heteroplasmy — where mitochondrial heteroplasmy is maintained in most individuals throughout a population, and the resultant heteroplasmic lineages potentially maintained through inheritance — is difficult to explain, particularly when much of the variation exists as synonymous mutations as is often observed in invertebrates (Robison et al., 2015).

One unexplored potential driver of unusual mitochondrial traits (e.g. heteroplasmy) is maternally-inheritance endosymbionts, such as the α‐proteobacteria genus *Wolbachia* which primarily act as maternally inherited reproductive parasites in most insect lineages. These endosymbionts can promote their transmission through a population by biasing for infected female offspring relative to uninfected individuals (Werren, 1997; Werren et al., 2008) through phenotypic alterations such as, (i) cytoplasmic incompatibility, (ii) feminization of genetic males, (iii) thelytokous parthenogenesis induction, and (iv) male killing; the mechanisms of which are poorly understood (Werren, 1997; Stouthamer et al., 1999). A consequence of the biased transmission of *Wolbachia* is that it can lead to rapid selection on host mitogenomes. These ‘selective mitochondrial sweeps’ occur when *Wolbachia*-infected host lineages replace non-infected linages and cause bacterially-facilitated dominance of the co-inherited mitogenome in the host population (Jiggins, 2003). Mitogenomes can ‘hitchhike’ their way to fixation and result in a host population with low mitochondrial haplotype variation (e.g. Ballad et al. (1996); Graham and Wilson (2012); Schuler et al. (2016)). Because mitochondria and *Wolbachia* are genetically linked through maternal transmission (Cariou et al., 2017), mitochondrial selective sweeps can occur when there are mechanisms that selectively favour *Wolbachia* (e.g. reproductive phenotypic manipulation) (Jiggins, 2003; Hurst and Jiggins, 2005). Consequently, selective mitochondrial sweeps are a major hurdle for studies of host demography that exclusively use mitochondrial markers (Jiggins, 2003; Charlat et al., 2009; Toews and Brelsford, 2012; Grant, 2015).

Here we use a variety of genetic assays to explore mitochondrial heteroplasmy in the bee *Amphylaeus morosus* (Colletidae: Hylaeinae) across its ∼2,000 km distribution along Australia's eastern coast (Houston 1975). We also co-examine the prevalence of two *Wolbachia* strains infecting *A. morosus* across its range and consider whether this dual infection may be related to the host's mitochondrial heteroplasmy.

## Methods

### Sampling

**Fig. 1:**
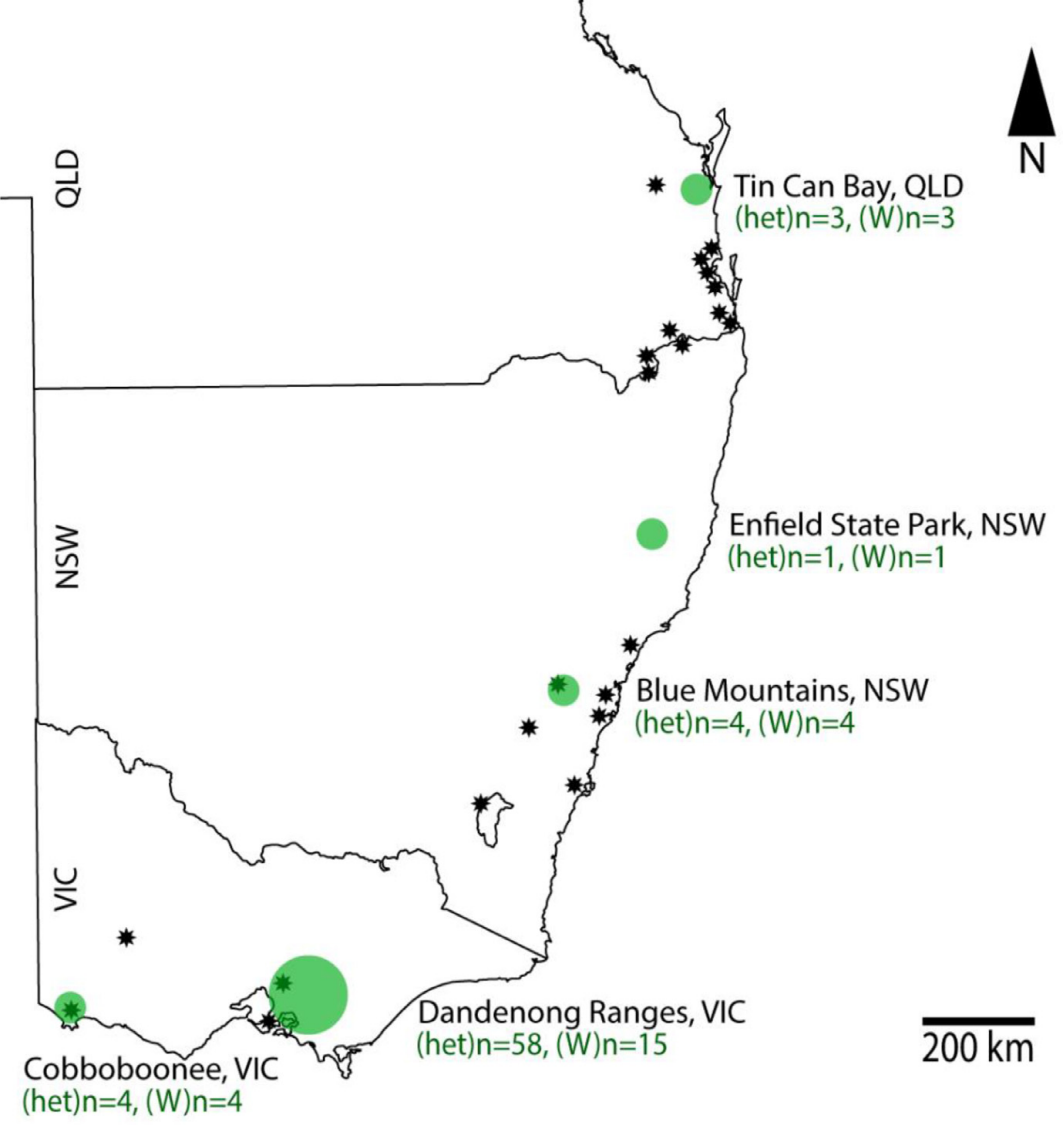
Recorded distribution (star points) of *Amphylaeus morosus* as reported by Houston (1975). Collection sites (green circles) for this study occurred across five locations of eastern Australia. Sample sizes of individuals barcoded for heteroplasmy in mt-COI region ((het)n) and *Wolbachia* infection ((W)n) from each location are included.

Intact colonies of *Amphylaeus morosus* were sampled from five regions along the eastern coast of Australia from southern Queensland (QLD), coastal New South Wales (NSW), and southwestern Victoria (VIC) (Fig. 1). From north to south, collections occurred in Tin Can Bay, QLD on 10^th^ December 2013, Enfield State Forest, NSW on 22^nd^ December 2018, Blue Mountains, NSW on 24^th^ July 2017, multiple collections from the Dandenong Ranges, VIC, and Cobboboonee State Forest, VIC (Fig. 1) on 22^nd^–24^th^ February 2017. At their geographical extremities, *A. morosus* were collected from dead flower scapes of *Xanthorrhoea* spp. in subcoastal heathland habitats. Specimens from the central localities were collected from dead abscised fronds of the tree fern *Cyathea australis* in wet montane forest. Intact nests were kept at 4°C until opening whereupon adults were transferred directly to 99% ethanol for preservation. Four collections from the Dandenong Ranges were conducted on 14^th^–18^th^ August 2014, 6^th^–10^th^ November 2014, 26^th^ February – 2^nd^ March 2015, and 21^st^–24^th^ November 2016. Immature individuals from the latter collection were reared in controlled conditions at Flinders University campus, South Australia to adulthood, to obtain adult males.

### DNA extraction

Total DNA was extracted from the tissue of a single hind leg from adult *A. morosus* specimens. Sixty-six females from across all regions and four males from the Dandenong Ranges were included (n = 70). Extractions used an adapted Gentra Puregene Cell Kit procedure (Qiagen, Chadstone VIC AUS) at the South Australian Regional Facility of Molecular Ecology and Evolution (SARFMEE) following manufacturers’ recommendations and were stored at 4°C.

### PCR and Sanger sequencing of mitochondrial DNA

PCR amplifications of mt-COI were carried out in a total volume of 25 µL, as follows: 1x MRT Buffer (1 X Immolase buffer/1.5mM MgCl2/0.8 mM dNTP mix/0.05 mg/ml BSA), primers (0.4 µM each), 1 U Immolase DNA Polymerase (Bioline, Eveleigh NSW, AUS), and 2 µL template DNA. The universal degenerate primer set COIF-PR115 and COIR-PR114 (Supp. Table 1) (Folmer et al. 1994) was used. PCR cycling conditions for specimens from the Tin Can Bay and Dandenong Ranges were one cycle (10 min at 95°C), 38 cycles (45 sec at 94°C, 45 sec at 48°C, 60 sec at 75°C), and one cycle (6 min at 72°C, 2 min at 25°C). For the remaining specimens, PCR conditions were one cycle (10 min at 94°C), five cycles (60 sec at 94°C, 90 sec at 45°C, 90 sec at 72°C), 35 cycles (60 sec at 94°C, 90 sec at 51°C, 60 sec at 72°C), and one cycle (10 min at 72°C, 2 min at 20°C). PCR amplified reaction products were visualised using 1.5% agarose gel. Successful PCR reaction products were purified using Multiscreen PRC384 Filter Plate (Millipore, Burlington MA USA) and re-suspended in 20–25 μL of 10 mM TRIS pH 8.0. Amplicons of 70 individuals were sent to the Australian Genome Research Facility (AGRF) in Adelaide, South Australia and sequenced using the ABI prism Big Dye Terminator Cycle sequencing kit with Applied Bio-Systems 3730 and 3730 xl capillary sequencers.

### Ion Torrent shotgun sequencing and mitogenome alignment for bee mtDNA

Total DNA was extracted from four legs and thoracic tissue of a single female bee (collected from the Dandenong Ranges in March 2015), using the DNeasy Blood and Tissue extraction kit (Qiagen), following the manufacturer's protocols. The sample (2.6 µg) was sequenced on an Ion Torrent PGM platform (Life Technologies) using a 318 chip with 400 bp chemistry using standard protocols at the AGRF facility. The DNA was sheared and a Pippin Prep (Sage Science, Beverly, MA, USA) was used to size-select fragments, to ensure most were greater than 300 bp and did not exceed 400 bp. Fragments were tagged by ligating with standard Ion Torrent barcode adaptors. Equal molar ratios of fragments from *A. morosus* and samples from unrelated projects were mixed prior to sequencing. Post sequencing fragments were demultiplexed, base called and aligned using Torrent Suite Software.

The resultant shotgun sequence fragments were mapped onto a *Hylaeus dilatatus* (Colletidae: Hylaeinae) reference mitogenome (Tan et al., 2015) (Genbank Accession: PRJNA274907) using the reference mapping function in Geneious version 10.2.2 (https://www.geneious.com) with the following optimized parameters for this dataset. Trimmed reads were mapped using Geneious’ low-sensitivity mapping function with a minimum mapping quality of ten, including flexibility for changes in fragment lengths compared to the reference genome, and minimum support for structural variation set to two reads. The alignment was then manually assessed and edited. Potential single nucleotide polymorphisms (SNPs), i.e. heteroplasmic sites, were identified using the Geneious SNP/Variation function (Supp. Table 1), ignoring sequence variation between *A. morosus* shotgun reads and the *H. dilatatus* reference genome. Geneious settings included a minimum read coverage set to six reads due to low read coverage, and a minimum frequency variant of 0.15. The maximum variant p-value and minimum strand-bias p-value were set to 10^−5^ with a 65% bias.

### *Wolbachia* assays across the host's distribution

*Wolbachia* screening was performed on genomic DNA extracts (leg tissue) previously used to assess mitochondrial heteroplasmy (i.e., all specimens analysed for *Wolbachia* infection were also assessed for mitochondrial heteroplasmy). All 70 individuals were firstly screened with 1.5% agarose gel electrophoresis of PCR products for *Wolbachia* COI-like *Wolbachia* gene region. This bacterial DNA gene region was reliably recovered using the broad arthropod mitochondrial COI primer sets below, a common problem with some arthropod barcoding primers (Smith et al., 2012). Of the 70 specimens, 27 individuals (24 females and 3 males) were selected for Sanger sequencing to assess co-infection of *Wolbachia* (Fig. 1). This included every individual from four of the regions (n = 12 individuals total) and a subset (n = 15 of 58 individuals) of those from collected from the Dandenong Ranges. PCR amplification protocols for the COI-like region of *Wolbachia* were performed using the arthropod (Lepidoptera) primer set Lep-F1 and Lep-R1 (Supp. Table 1) (Hebert et al., 2004) for most specimens, as well as the universal primers LCO1490 and HCO2198 (Supp. Table 1) (Folmer et al., 1994) for five specimens.

PCR amplifications of the *Wolbachia* COI-like region were carried out in a total volume of 25 µL, as above. PCR cycling conditions were one cycle (10 min at 95°C), 35 cycles (45 sec at 94°C, 45 sec at 48°C, 60 sec at 75°C), and one cycle (6 min at 72°C, 2 min at 25°C). PCR products were visualised using 1.5% agarose gel. Successful PCR products were purified and sequenced at AGRF.

### Cloning to identify *Wolbachia* strains

To distinguish between the *Wolbachia* strains involved in this co-infection, one individual each from the northern- and southern- most localities (Tin Can Bay, QLD and Dandenong Ranges, VIC) was selected for cloning. These specimens had previously been Sanger sequenced and their chromatograms had indicated co-infections of two *Wolbachia* strains. For comparison with our other *Wolbachia* data, we produced clones for the *Wolbachia* COI-like region, as well as *Wolbachia* surface protein (*wsp*) gene region (see below). The COI-like region was recovered using the “Lep” primer set (Hebert et al., 2004) and PCR amplification conditions were repeated from the Sanger sequencing above. Four additional *Wolbachia* COI-like sequences were recovered from cloning products using the universal degenerate primer set COIF-PR115 and COIR-PR114 (Supp. Table 1) (Folmer et al., 1994) which were originally intended to recover bee mt-COI (cloning was not able to recover host mtDNA).

The *wsp* gene was also analysed as it is one of the fastest evolving *Wolbachia* genes and can provide a good indication of phylogenetic relationships among closely related *Wolbachia* strains (Zhou et al., 1998). The primer set wsp-81F and wsp-691R (Supp. Table 1) (Braig et al., 1998; Zhou et al., 1998) was used and PCR conditions followed Zhou et al. (1998). All reactions were carried out in PCR volumes as described above.

All amplified DNA was purified and PCR products 1 μL neat from the COI-like gene with Hebert et al. (2004) primers and 2 μL neat from the *wsp* gene with Zhou et al. (1998) primers were cloned into pGEM-T Easy (Promega, Madison, USA) and blue/white screening following manufactures instructions. Transformants were harvested from Petri dishes and suspended in 25 mL of 10 mM TRIS and then heat-treated to lyse cells. All colonies were amplified using the primer set T7 and SP6 (Supp. Table 1) (Promega) and the following PCR conditions: one cycle (10 min at 95°C), 34 cycles (30 sec at 94°C, 30 sec at 60°C, 90 sec at 72°C), and one cycle (20 min at 72°C). Purified cloned amplicons were sequenced (Sanger) at AGRF.

### Analyses of Sanger sequences

Sequencing editing and alignment was completed using Geneious for the (i) bee mt-COI sequences, (ii) *Wolbachia* COI-like sequences, and (iii) cloning products of the *Wolbachia* COI-like and *wsp* genes. To confirm the identity of the source species for each sequence, edited sequences were BLAST-screened (Altschul et al., 1990) against the National Center for, 1988 database (http://www.ncbi.nlm.nih.gov/blast).

### Data availability

All sequencing data, including Sanger sequences of mitochondrial COI, *Wolbachia* COI-like and *Wolbachia* cloning data, Ion Torrent shotgun mitogenome alignment, and phylogenetic analyses of *Wolbachia* genes (Supp. Figs. 5–7) is available via https://doi.org/10.25451/flinders.19193519.v1.

## Results

### Chromatogram evidence of heteroplasmy

**Fig. 2:**
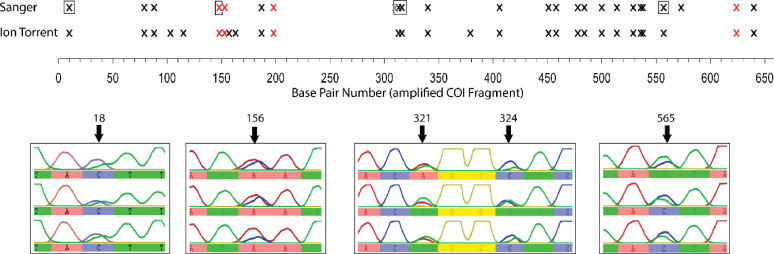
Summary of mt-COI heteroplasmic sites found using two techniques; Sanger sequencing and Ion Torrent shotgun sequencing. Black crosses represent nucleotide substitutions that were synonymous, and red crosses represent nucleotide substitutions that were non-synonymous (amino acid changing). An additional synonymous nucleotide was detected at 310 bp in an individual from the Blue Mountains, New South Wales (in grey). The chromatograms below are examples of the double peaks observed in the chromatogram of Sanger sequences. Their nucleotide position in the COI fragment is indicated by the sites encapsulated in boxes in the technique summary, which correspond to the numbers above the chromatograms.

We obtained a total of 70 forward mt-COI fragments (where four of these specimens were also re-sequenced to examine reproducibility of results) and reverse mt-COI Sanger sequences were produced for 13 of these specimens (sequence lengths ranging from 613 bp to 658 bp). Every specimen (both sexes) exhibited double peaks at specific sites throughout the mt-COI fragment. These heteroplasmic sites were identified in individuals from all populations and in the resequenced samples, implying that these mitochondrial haplotypes are shared across the entire *A. morosus* range. For this reason, we classified all ‘shared’ double peaks (observed in most individuals from *all* geographic regions) as stable heteroplasmic sites across the species. In total, 24 stable heteroplasmic sites were consistently recovered across the 658 bp mt-COI fragment (Fig. 2). An additional double peak was detected in a single specimen from the Blue Mountains, NSW (position 310 bp; Fig. 2), resulting in a novel synonymous-coding heteroplasmic site.

Mitochondrial protein translations of heteroplasmic sites indicated four non-synonymous changes — detected via Sanger sequencing (Fig. 2) — none of which involved mitochondrial stop codons. An uncorrected ‘p’ pairwise distance (i.e. percentage difference in nucleotide sites) for the two 658 bp mt-COI strain sequences, calculated using Geneious, gave a minimal sequence divergence of 3.8%, considering both synonymous and non-synonymous changes.

### Nucleotide variation throughout the mitogenome

**Fig. 3:**
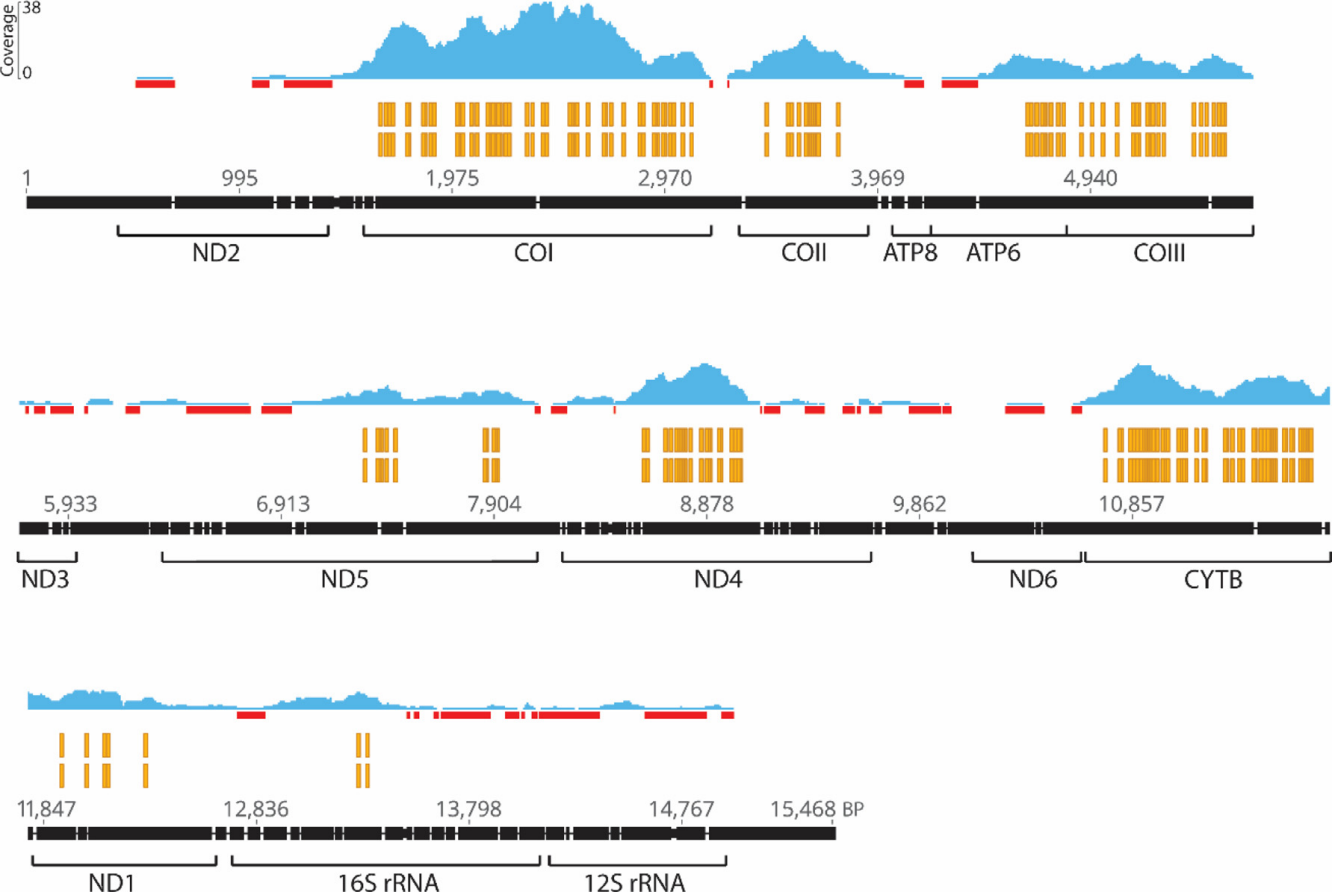
*Amphylaeus morosus* Ion Torrent shotgun sequencing alignment to the *Hylaeus dilatatus* reference mitogenome (Tan et al. 2015). The locations of the major protein-coding and rRNA genes of *H. dilatatus* mitochondrial genome are shown. Read coverage (blue) represents the comparative amount of the *A. morosus* shotgun contigs that aligned to the reference genome and red bars indicate where coverage was only a single contig. Using the Geneious version 10.2.2 SNP/Variation function, 214 variable sites were identified, indicated by the yellow markers, and suggests widespread heteroplasmic sites throughout the mitogenome.

The Ion Torrent shotgun run produced a total of 782,607 reads and were aligned to the *H. dilatatus* mitogenome (Tan et al., 2015). Initially, 5,151 contigs aligned to the *H. dilatatus* mitogenome. After post-examination and editing, 466 high quality contigs were retained. Most contigs aligned to protein-coding regions, but with some poorly-matched alignments to the two rRNA regions, 16S, and 12S rRNA (Fig. 3).

These data indicated that heteroplasmic sites are distributed throughout the mitogenome of *A. morosus*; all mitochondrial genes with aligned contigs contained heteroplasmic sites. A total of 214 heteroplasmic sites were identified using the Geneious SNP/Variation function (Fig. 3), consisting entirely of single nucleotide substitutions (i.e., no insertions, deletions, or tandem repeats). All of the heteroplasmic sites detected by the Geneious SNP/Variant function involved only two base pairs (Fig. 3).

A comparison of the two sequencing techniques (Sanger and Ion Torrent) demonstrated that heteroplasmic sites were, with few exceptions, consistently recovered (Fig. 2, Supp. Table 3). An additional four variable sites were identified in the mt-COI region with Ion Torrent. The Ion Torrent platform has been shown to have a higher SNP call of true positives, but also higher call of false positives, compared to the Illumina platform (Quail et al., 2012). However, given that all but one of the 24 (Sanger) heteroplasmic sites in mt-COI were otherwise recovered using both techniques, we interpreted our NGS data as being generally reliable; though it might be slightly overestimating SNP variation.

### Widespread co-occurring *Wolbachia* infection

For all 70 individuals, positive PCR products were observed through gel electrophoresis for the *Wolbachia* COI-like gene (indicating all individuals were infected with at least one strain of *Wolbachia*). To explore further for evidence of co-infection prevalence, sequences were produced for 27 *A. morosus* specimens for the *Wolbachia* COI-like gene (567 bp to 613 bp) which was clearly distinguished from host mitochondrial DNA based on BLAST results. All specimens sequenced for *Wolbachia* (n = 27) from every locality were observed to have a co-infection of *Wolbachia* as they showed clear double peaks at consistent nucleotide sites in their chromatograms (Supp. Fig. 4) suggesting a persistent co-infection of two *Wolbachia* strains across the host's distribution. Cloning data recovered two haplotypes for both *Wolbachia* genes examined, indicating a consistent *Wolbachia* co-infection in *A. morosus,* extending from Tin Can Bay (northern) and Dandenong Ranges (southern). When aligned to the COI-like Sanger sequences, double peaks in the chromatograms occurred at variable sites detected through cloning (e.g. Supp. Fig. 4).

## Discussion

Our analyses of *Amphylaeus morosus* indicated three unusual phenomena: (i) the widespread prevalence of mt-COI heteroplasmy restricted to consistent nucleotide sites, and which shotgun sequencing data from a single individual suggested extends to multiple protein-coding genes in the mitogenome, (ii) a near-complete lack of mtDNA variation within both mitogenomes over a very large geographical range (∼2,000 km), and (iii) a consistent co-infection by two supergroup A *Wolbachia* strains across the host's range.

### Widespread and conserved mitochondrial heteroplasmy

In insect studies, reports of mitochondrial heteroplasmy are mostly reported for the widely sequenced mitochondrial ‘barcode’ region “COI” because of its prevalent use in systematic studies (De Mandal et al., 2014; Hendrich et al., 2011; Shokralla et al., 2014). Whole mitogenome exploration of heteroplasmy is not yet widely performed in insects. Within Hymenoptera, mitochondrial heteroplasmy has been almost exclusively reported from bees (with a notable report in ants (Meza-Lázaro et al., 2018)). At least 29 bee species from five of the seven bee families have been identified with unusually high rates of mitochondrial heteroplasmy (Magnacca and Brown, 2010; Magnacca and Brown, 2012; Françoso et al., 2016; Ricardo et al., 2020b; Songram et al., 2006), but the most extensively studied group are the *Hylaeus* of Hawai'i (Magnacca and Brown, 2010).

For our study, uniform heteroplasmic sites across individuals and localities are suggestive of widespread heritable mitochondrial heteroplasmy retained across generations. Additionally, Ion Torrent shotgun sequencing data obtained from one female, further suggested extensive heteroplasmic sites (>200 SNPs) throughout the mitogenome. This is an unparalleled number of mitochondrial variable sites compared with other invertebrate heteroplasmic systems (Xiong et al., 2013; Sriboonlert and Wonnapinij, 2019; Meza-Lázaro et al., 2018; Moreira et al., 2017). High divergences between the heteroplasmic mitogenomes (i.e., 3.8% at COI) might initially suggest that one mitogenome was introduced in the past, perhaps following a hybridization event. However, analyses of Hawaiian *Hylaeus* (a genus closely related to *Amphylaeus*) suggests that hylaeine bees may be predisposed to high levels of mitochondrial divergence (Magnacca and Danforth, 2006). Additionally, there are numerous reports that heteroplasmic individuals can possess several mitochondrial haplotypes (Magnacca and Brown, 2010) (e.g. an average of seven mtDNA haplotypes per heteroplasmic individual of *Bombus morio* (Hymenoptera: Apidae) (Ricardo et al., 2020a)). Our analyses indicated two mtDNA lineages in *A. morosus*, with some evidence of intra-individual variations within these lineages (Supp. Fig. 1–4).

We suggest that our data are best-explained by widespread and consistent mitochondrial heteroplasmy, although nuclear inclusions of mitochondrial genes (NUMTs) is a possible alternative (Magnacca and Brown, 2010; Ricardo et al., 2020a; Cihlar et al., 2020). We reason that heteroplasmy is most likely because (i) whole-mitogenome transferrals into the nucleus are very rare, (ii) no mitochondrial stop codons were associated within any heteroplasmic sites examined for four mtDNA gene isolates in the Ion Torrent data (Supp. Figs. 1–4), (iii) examination of Ion Torrent gene isolates showed that a similar number of contigs were sorted into each mtDNA ‘lineage’ (Supp. Figs. 1–4) suggesting that they occurred at a similar frequency, inconsistent with one of the sources being from a single nuclear DNA insert, and (iv) it would require the ‘NUMT’ spreading over an enormous geographical range without acquiring additional mutations. However, the maintenance of consistent mitochondrial heteroplasmy across the species’ very wide distribution is puzzling.

The effective population size of mtDNA during embryogenesis is very small, with estimates being a few hundred copies per cell in the early stages of primordial germ cell production (Jansen, 2000; Cao et al., 2007; Cree et al., 2008). Small effective population size should lead to strong genetic drift, such that if two mitogenomes were present at any one time, either could be rapidly lost across multiple cell-division cycles. Consequently, if there were indeed two divergent mitogenomes present in *A. morosus*, there must be some mechanism(s) that operates to maintain their dual presence in a way that counteracts genetic drift. That same mechanism might be maintaining the near-absence of inter-individual variation. This species is sexually determined by haplodiploidy (where males are produced from unfertilized eggs which means they are produced with no paternal contribution). Therefore, because *A. morosus* males are also heteroplasmic, mitochondrial heteroplasmy must be maternally inherited in this species. Maternal inheritance of mitochondrial heteroplasmy has also been proposed for the ant, *Ectatomma ruidum* (Hymenoptera: Formicidae) (Meza-Lázaro et al., 2018); however, for other Hymenoptera and haplodiploid groups, the mechanisms that might enable heteroplasmy to persist with populations are still unknown (e.g. Gawande et al. (2017); Magnacca and Brown (2010); Magnacca and Brown (2012)).

### Distribution-wide *Wolbachia* co-infection

In addition to widespread mitochondrial heteroplasmy, *A. morosus* individuals were observed to also be consistently infected with two supergroup A *Wolbachia* strains in samples across its entire range (Supp. Fig. 4). *Wolbachia* infections occur frequently in bees, with supergroup A strains being the most common (Gerth et al., 2013; Gerth et al., 2011). Co-infections of different *Wolbachia* strains are also reported within bees (Gerth et al., 2011; Gerth et al., 2013; Jeyaprakash et al., 2009). But like other insect taxa, attempts to identify endosymbiont infections within bees has been generally limited to broad and general sampling within narrow geographical areas of interest (with low intra-specific sample sizes), or within specific taxa (often those with close ties to human activities). Therefore, the biological roles of these endosymbionts within bee hosts have not yet been explored.

Both *Wolbachia* strains identified in *A. morosus* have close relatives that cause reproductive phenotype modifications within their respective hosts (Sasaki and Ishikawa, 1999; Charlat et al., 2002; Sasaki et al., 2002; Sasaki et al., 2005). BLAST results indicated that one of the *Wolbachia* strains was most closely related to the *w*Ha (hosted in *Drosphila simulans*; (Ellegaard et al., 2013)) and *w*CauA (hosted in the pyralid moth *Cadra cautella*; (Sasaki and Ishikawa, 1999)) strains; therefore, we labelled this *A. morosus* strain in *w*AmHa. The second strain did not closely match any published *Wolbachia* genome, therefore we labelled this strain *w*Amor. Interestingly, close relatives of *w*AmHa commonly co-occur with other *Wolbachia* strains (Poinsot and Merçot, 1997; Sasaki and Ishikawa, 1999). It is feasible that in *A. morosus*, either/both strains could have induced a reproductive alteration in order to reach infection fixation across the *A. morosus* distribution. Co-infections are reasonably common (Duron et al., 2008), and could represent a transitional phase of strain replacement or stable co-infection. Additionally, there is some evidence that stable co-infections can increase the fitness of the infecting *Wolbachia* strains (Driscoll et al., 2020).

Our analyses indicated the two *Wolbachia* strains in *A. morosus* diverged ∼61.5 mya (Supp. Figs. 5–7) which predates the origin of the Hylaeinae (Almeida and Danforth, 2009). Hence it is unlikely that these two bacterial strains diverged within *A. morosus*, rather they probably entered this host independently. For novel strains to independently infect a host lineage, *Wolbachia* needs to be transmitted via either horizontal or paternal transmission (Keeling et al., 2003). Our results concur with scenarios raised by Bailly-Bechet et al. (2017) where most *Wolbachia* infection events have occurred within the last few million years. Furthermore, we identified minor genetic variation within each strain between the geographical localities. Divergence time estimates of these data indicated that *w*Amor has been diverging within *A. morosus* for ∼2 mya (95% HPD = 0.3, 4.1 mya; Supp. Fig. 5), whereas *w*AmHa has been diverging for ∼0.5 mya (95% HPD = 0, 1.3 mya; Supp. Fig. 5), although it should be noted that the 95% HPDs overlap. However, this provides some support that there was not a single horizontal transfer event of both *Wolbachia* strains from a different co-infected host species. Further analysis of genetic variation with this *Wolbachia* co-infection across geographic distribution of *A. morosus* would help us to understand the mechanisms of the proposed transmission event(s).

### *Wolbachia*’s proposed influence on *Amphylaeus morosus* mtDNA

**Fig. 4:**
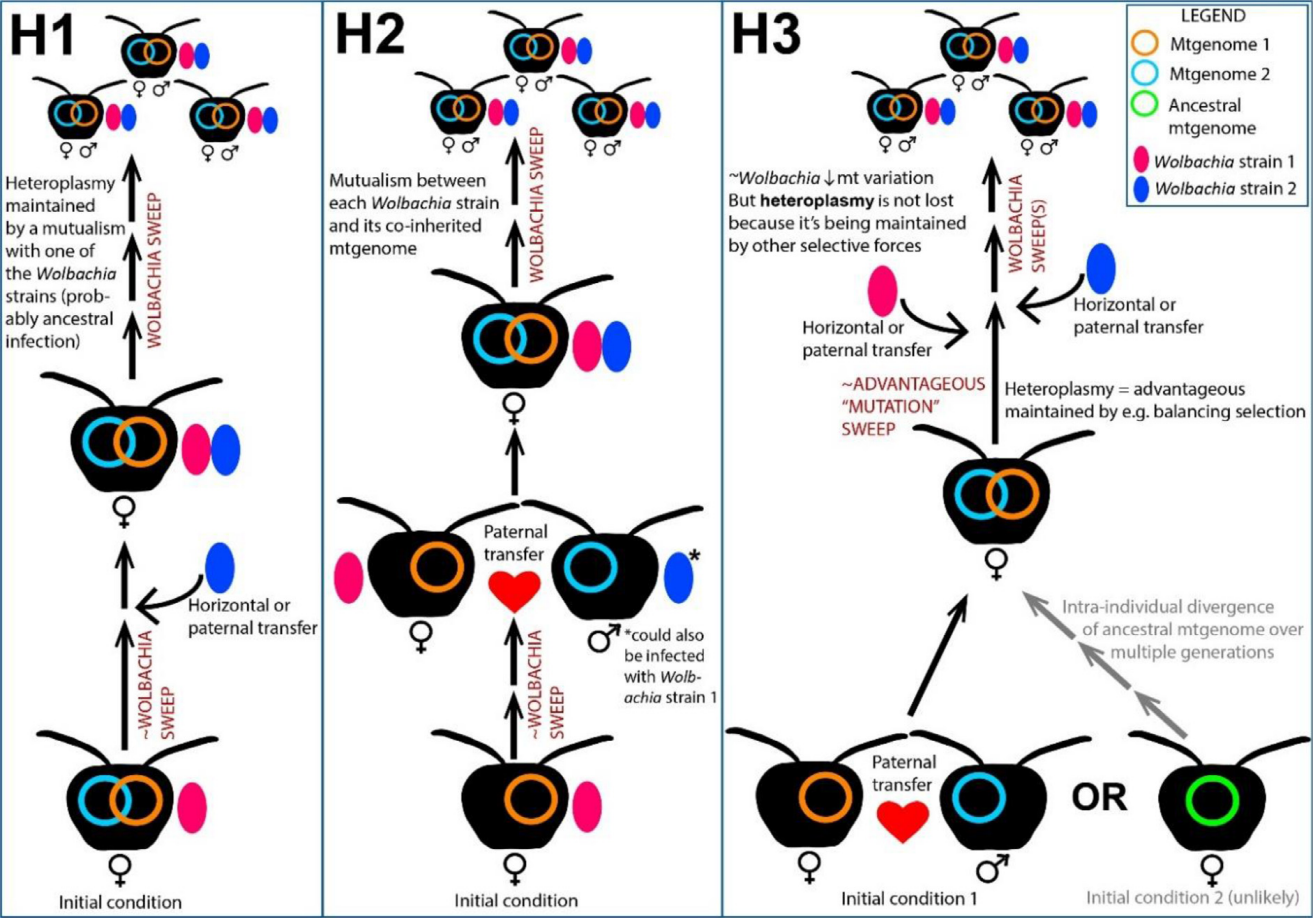
Three proposed hypotheses that could explain the widespread and consistent co-occurring heteroplasmic mitogenomes and *Wolbachia* strains in *Amphylaeus morosus*: H1: The heteroplasmic founder hypothesis; H2: The co-inheritance hypothesis; and H3: The null-*Wolbachia* hypothesis.

It is likely that the mtDNA patterns observed in *A. morosus* have been influenced by one or both *Wolbachia* strains for the following reasons. Firstly, the extremely low mtDNA diversity across its entire ∼2,000 km range is concordant with a *Wolbachia*-induced selective sweep (Jiggins, 2003; Schuler et al., 2016). Secondly, relatives of both *Wolbachia* strains infecting *A. morosus* are known to have host reproductive modification potential (Sasaki et al., 2002; Sasaki et al., 2005; Charlat et al., 2002) which provides further support for the potential of these strains to induce a selective sweep. Thirdly, it is doubtful that a recent population bottleneck is a sufficient explanation for the lack of mtDNA variation, given the large geographical distribution of *A. morosus* (Houston, 1975). A *Wolbachia*-induced selective sweep might explain the lack of mtDNA variation however, the potential mechanism(s) that preserve widespread, consistent, and heritable heteroplasmy remain unclear. To address this, we propose several hypotheses that might explain the widespread fixation and maintenance of conserved heteroplasmic mitochondria in *A. morosus* (Fig. 4).

(i) **The heteroplasmic founder hypothesis (H1)**: An *A. morosus* individual already possessed two mitogenomes (was heteroplasmic) and a single *Wolbachia* infection (which could have already swept through the population). A second *Wolbachia* strain entered this lineage (via horizonal or paternal transfer) and spread to fixation within the population, dragging along both mitogenomes and the initial *Wolbachia* strain. Mitochondrial heteroplasmy is maintained because of a mutualism with one of the *Wolbachia* strains (probably the ancestral infection).
(ii) **The co-inheritance hypothesis (H2)**: Two *A. morosus* individuals with different *Wolbachia* strains mated (each with a single divergent mitogenome), and both mitogenomes and *Wolbachia* strains were maintained in their progeny via paternal transmission of both a mitochondrial and bacterial genome. These co-occurring *Wolbachia* strains and their co-inherited mitogenomes were then swept (via maternal inheritance) through the population to fixation. Each mitogenome is maintained by a mutualism with its corresponding *Wolbachia* strain.
(iii) **The advantageous heteroplasmy hypothesis (H3)**: The maintenance of mitochondrial heteroplasmy in *A. morosus* is unrelated to either *Wolbachia* strain. A selective sweep via either *Wolbachia* and/or an advantageous mutation (heteroplasmy) could be responsible for the overall loss of mitochondrial diversity. However, one of the two mitogenomes was never lost (post-fixation) because heteroplasmy is advantageous over homoplasmy.

Our hypotheses include many assumptions about the biology of the host and parasite, and we cannot confirm which and or either hypothesis applies to our system, or to similar systems. *Wolbachia* is a strong candidate for the mechanism maintaining the unusual mtDNA traits observed in *A. morosus.* However, other explanations remain. Maintenance of widespread heteroplasmy could be caused by other selective processes, such as mechanisms similar to heterozygote advantage as seen in nuclear genomes and/or positive or balancing selection. For example, in *Drosophila* it has been demonstrated that some mtDNA haplotypes have selective advantages when host individuals are exposed to different thermal conditions (Camus et al., 2017; Lajbner et al., 2018). These patterns of temperature-dependant selection have also been demonstrated in other taxa, such as the seed beetle *Callosobruchus maculatus* (Coleoptera: Chrysomelidae) (Immonen et al., 2020) and yeasts in the genus *Saccharomyces* (Baker et al., 2019). *Amphylaeus morosus* occupies areas susceptible to extreme temperature ranges; for example, temperatures in the Dandenong Ranges, VIC, can range between from -2.7°C to 46.1°C (Bureau of Meteorology, 2020). However, in other regions that have lower seasonal temperature variation, such as sub-tropical heathlands, the maintenance of heteroplasmy via temperature-dependent selection seems less likely. Given these two mtDNA haplotypes are present across the entire distribution of *A. morosus*, the selective driver maintaining heteroplasmy is likely operating in all habitats and climatic conditions. Identifying such a driver (beyond the hypothesized *Wolbachia*) is challenging. Each hypothesis will likely require extensive manipulative or modelling experiments to test. However, *A. morosus* presents a unique opportunity to investigate these complex interactions.

## Declaration of Competing Interest

None.

## Appendix Supplementary materials

Image, application 1
