# Supplementary material for: Unparalleled mitochondrial heteroplasmy and Wolbachia co-infection in the non-model bee, Amphylaeus morosus
Source: Curr Res Insect Sci. 2022 Apr 20;2:100036. doi: 10.1016/j.cris.2022.100036 (PMC9387454; doi:10.1016/j.cris.2022.100036)
Supplement: Supplementary file 1 [file mmc1.docx]

**Supplementary Material**

Supp. Table 1: Primer sets used to generate both host mitochondrial and *Wolbachia* loci for DNA analyses.

| **Locus Amplified** | **Primer Name** | **Sequence** |
| --- | --- | --- |
| mt-COI | COIF-PR115  COIR-PR114 | 5'-TCWACNAAYCAYAARGAYATTGG-3'  5'-ACYTCNGGRTGNCCRAARARYCA-3' |
| *Wolbachia* COI-like | LCO1490  HCO2198 | 5’-GGTCAACAAATCATAAAGATATTGG-3’  5’-TAAACTTCAGGGTGACCAAAAAATCA-3’ |
| *Wolbachia* COI-like | Lep-F1  Lep-R1 | 5'-ATTCAACCAATCATAAAGATAT-3'  5'-taaacttctggatgtccaaaaa-3' |
| *Wolbachia wsp* | wsp-81F  wsp-691R | 5’-TGGTCCAATAAGTGATGAAGA-3'  5'-AAAAATTAAACGCTACTCCA-3' |
| Cloning products | T7  SP6 | 5'-TAATACGACTCACTATAGGG-3'  5'-TTCTATAGTGTCACCTAAAT-3' |

**Supp. Material A: Mitochondrial gene isolation of shotgun sequences**

**A. 1. Methods:** Four mitochondrial genes were isolated from the aligned Ion Torrent shotgun sequences produced from the female *A. morosus* specimen: cytochrome *c* oxidase subunit I (mt-COI), cytochrome *c* oxidase subunit II (mt-COII), cytochrome b (mt-cytb), and NADH-ubiquinone oxidoreductase chain 4 (mt-ND4). These genes had the highest read depths (>15 contigs) and were individually isolated from the alignment to be examined thoroughly for evidence of recombination, NUMTs, and ambiguities.

Conducted in two steps, the aligned contigs within these four genes were firstly reduced to show only suspected heteroplasmic sites (i.e. all non-variable sites within the contigs were hidden) (Supp. Table 2, Supp. Figures 1–4). The (aligned) heteroplasmic sites retained for assessment included both those detected by the Geneious SNP/Variation function in Geneious version 10.2.2 (<https://www.geneious.com>) and those that were manually observable but were not detected. This is because the Geneious SNP/Variation function demonstrated a bias against detecting potential heteroplasmic sites that included a T nucleotide, even if they occurred at the same frequency as other nucleotide combinations across the aligned contigs. A lower quality score assigned to T nucleotides by Ion Torrent (which is a factor the Geneious SNP/Variation detection function includes when assessing potential variable sites) could be responsible for this apparent bias. This reduced the length of the gene fragments by ~90–97%, so that contigs could be sorted into analogues based on variable sites only (Supp. Table 2, Supp. Figures 1–4). An assessment of the number of ‘lineages’ (i.e. the number of types of mitogenomes or possible NUMTs) that were present within the sample for each of the four genes was then evaluated. Secondly, these aligned, analogous contigs were then assessed in their entirety (i.e. non-variable sites were included) and the mitochondrial protein translation scheme was applied to investigate whether there were any mitochondrial stop codons which could indicate NUMTs, rather than multiple mitogenomes.

**A. 2. Results:** For mitochondrial fragments isolated from the shotgun alignment (genes with the highest read depth, viz. mt- COI, COII, cytb, and ND4), analogue contigs were sorted into two common, separate ‘lineages’ where adjacent heteroplasmic sites were generally not shared between lineages. Within each lineage, contigs could be further separated by within-lineage variation into ‘groups’ (Supp. Figures 1–4), however, these within-lineage differences presented as minor variation (dissimilar in 1–3 consecutive variable nucleotides, see arrows in Supp. Figures 1–4). The source of this within-lineage variation is unknown, but it could be due to recombination (see Supp. Figure 3 for an example), naturally occurring variation due to mutagenetic processes, or sequencing error. Further next-generation sequencing efforts to produce datasets across multiple individuals at greater read depth is recommended to address these variable sites more thoroughly. Some additional contigs (usually present as singletons; Supp. Figures 1–4) were recovered that could not be assigned to either of the two main lineages. These could be the result of NUMTs (nuclear inclusion of mtDNA), additional minor mtDNA variants, or sequencing error, however these data are not extensive enough to determine their true origins.

Mitochondrial protein translations (using the invertebrate mitochondrial coding scheme) for each of the four mitochondrial gene fragments did not reveal mitochondrial stop codons at heteroplasmic sites within either of the lineages, which would suggest one lineage is due to a NUMT. However, stop codons were detected earlier in the reading frame of the gene isolates (both lineages) than would be expected compared to the *H. dilatatus* reference mitogenome (Tan et al., 2015). When these stop codons occurred in a gene, they were positioned in homologous locations in both mitochondrial lineages and succeeded AT rich regions with homopolymers, where Ion Torrent sequencing is known to be prone to errors (Quail et al., 2012, Bragg et al., 2013). Adjusting these homopolymer regions by changing the length by one base always shifted the stop codon so that it occurred at the approximately expected position in the gene.

Supp. Table 2: The number of contigs recovered for each of the Ion Torrent mitochondrial gene isolates examined. Total length of aligned contigs were reduced to only show suspected SNPs (variable nucleotide sites indicating heteroplasmy). The number of SNPs detected by the Geneious "SNP/Variation" function are also reported. By isolating only the SNP’s to sort contigs into lineages, the gene fragments were reduced by 90–97% of their original length. Note: once the lineages were established, the full lengths of the gene isolates were restored to assess for coding changings associated with variable sites.

| **Gene** | **No. contigs** | **Max. contig depth** | **Total fragment length** | **All suspected SNPs** | **Geneious SNPs only** | **Fragment length reduction** |
| --- | --- | --- | --- | --- | --- | --- |
| COI | 150 | 38 | 1,610 | 51 | 51 | 97% |
| COII | 34 | 21 | 668 | 69 | 25 | 90% |
| cytb | 52 | 19 | 1,224 | 81 | 58 | 93% |
| ND4 | 32 | 20 | 572 | 27 | 24 | 95% |

Supp. Table 3: Summary of heteroplasmic sites detected in mitochondrial cytochrome c oxidase subunit 1 (COI) gene fragment using both techniques: Sanger sequencing and Ion Torrent shotgun sequencing. Base pair (bp) positions are in reference to the combined Sanger sequences (forward + reverse sequence) which was 658 bp in length. A heteroplasmic site detected at 310 bp was identified in a single individual (AM6) from the Blue Mountains, NSW. The heteroplasmic site at 624 bp was observed in the Sanger sequence chromatograms, but was observed as within lineage variation (minor variation) within the Ion Torrent shotgun data.

| **Base Pair (bp)** | **Sanger** | **Ion Torrent** | **Allele 1** | **Allele 2** | **Non-synonymous** | **Amino Acid** |
| --- | --- | --- | --- | --- | --- | --- |
| 10 | ✓ | ✓ | C | T | 🗶 | Y |
| 79 | ✓ | ✓ | T | A | 🗶 | S |
| 88 | ✓ | ✓ | C | T | 🗶 | N |
| 103 | 🗶 | ✓ | T | C | 🗶 | N |
| 115 | 🗶 | ✓ | T | C | 🗶 | Y |
| 148 | ✓ | ✓ | A | C | ✓ | **M-I** |
| 153 | ✓ | ✓ | C | T | ✓ | **S-F** |
| 157 | 🗶 | ✓ | C | T | 🗶 | F |
| 163 | 🗶 | ✓ | T | C | 🗶 | V |
| 187 | ✓ | ✓ | C | T | 🗶 | F |
| 198 | ✓ | ✓ | G | T | ✓ | **W-L** |
| 310 | *AM6 (NSW) only* | 🗶 | C | T | 🗶 | Y |
| 313 | ✓ | ✓ | T | A | 🗶 | T |
| 316 | ✓ | ✓ | T | C | 🗶 | G |
| 340 | ✓ | ✓ | T | C | 🗶 | Y |
| 379 | 🗶 | ✓ | G | A | 🗶 | S |
| 406 | ✓ | ✓ | T | C | 🗶 | H |
| 451 | ✓ | ✓ | A | T | 🗶 | V |
| 458 | ✓ | ✓ | C | T | 🗶 | L |
| 478 | ✓ | ✓ | C | T | 🗶 | N |
| 484 | ✓ | ✓ | C | T | 🗶 | N |
| 500 | ✓ | ✓ | T | C | 🗶 | L |
| 514 | ✓ | ✓ | A | T | 🗶 | A |
| 529 | ✓ | ✓ | C | T | 🗶 | A |
| 536 | ✓ | ✓ | C | T | 🗶 | L |
| 538 | ✓ | ✓ | T | A | 🗶 | L |
| 557 | ✓ | ✓ | C | T | 🗶 | L |
| 573 | ✓ | 🗶 | C | G | 🗶 | T |
| 624 | ✓ | ~ (minor) | G | A | ✓ | **G-D** |
| 640 | ✓ | ✓ | C | T | 🗶 | I |
| TOTAL | 24 | 28 |  |  | 4 |  |


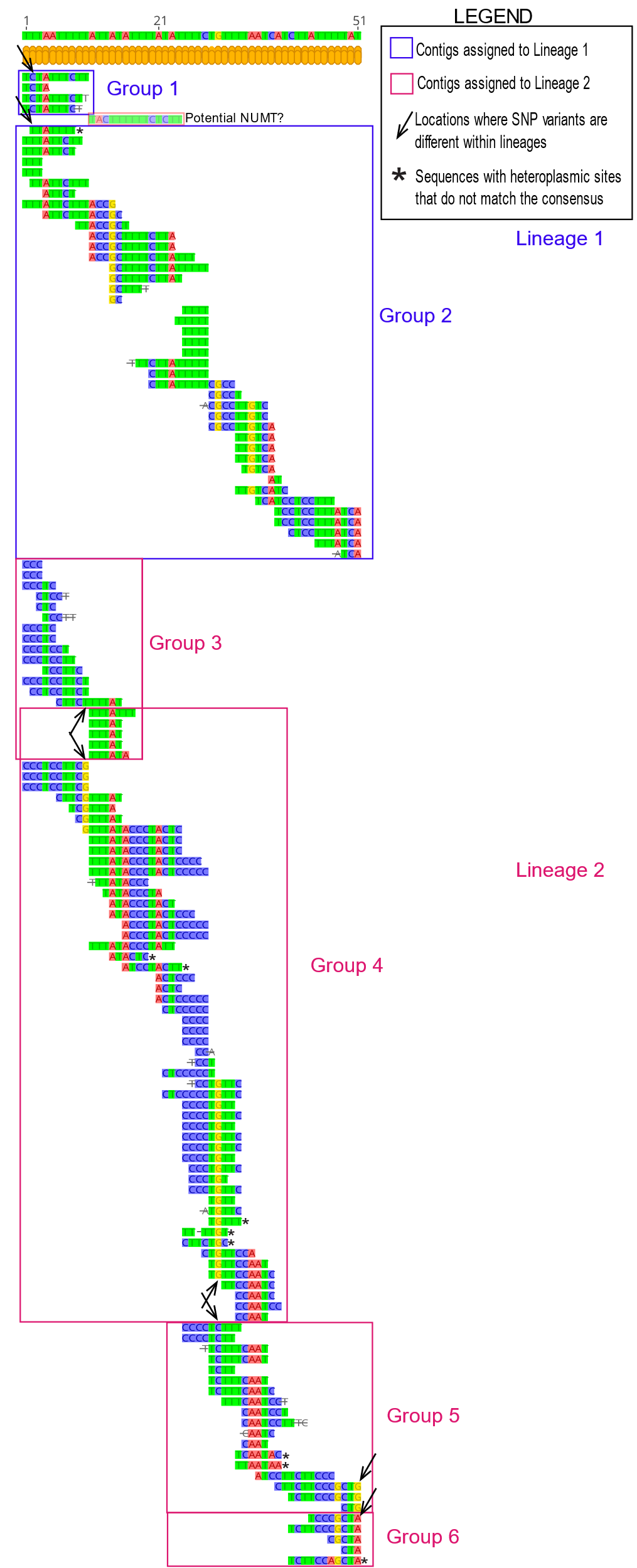


Supp. Figure 1: Ion Torrent shotgun sequence alignment of suspected heteroplasmic sites in the mitochondrial cytochrome c oxidase subunit I (COI) gene. It is important to note that the contigs presented here only consist of variable sites and have been reduced in length by ~90% of their original length (~150 – 500 bp per contig). Almost all of these variable sites are separated by long, non-variable regions shared across the dataset. These reduced-contigs have been sorted into analogues producing the two proposed heteroplasmic “lineages”. Within lineages, variable nucleotide sites have been indicated by an arrow and these contigs were classified into “groups”. Some reduced-contigs were not able to be sorted into any lineage and are suspected to be NUMTs, additional mtDNA variants, or errors.


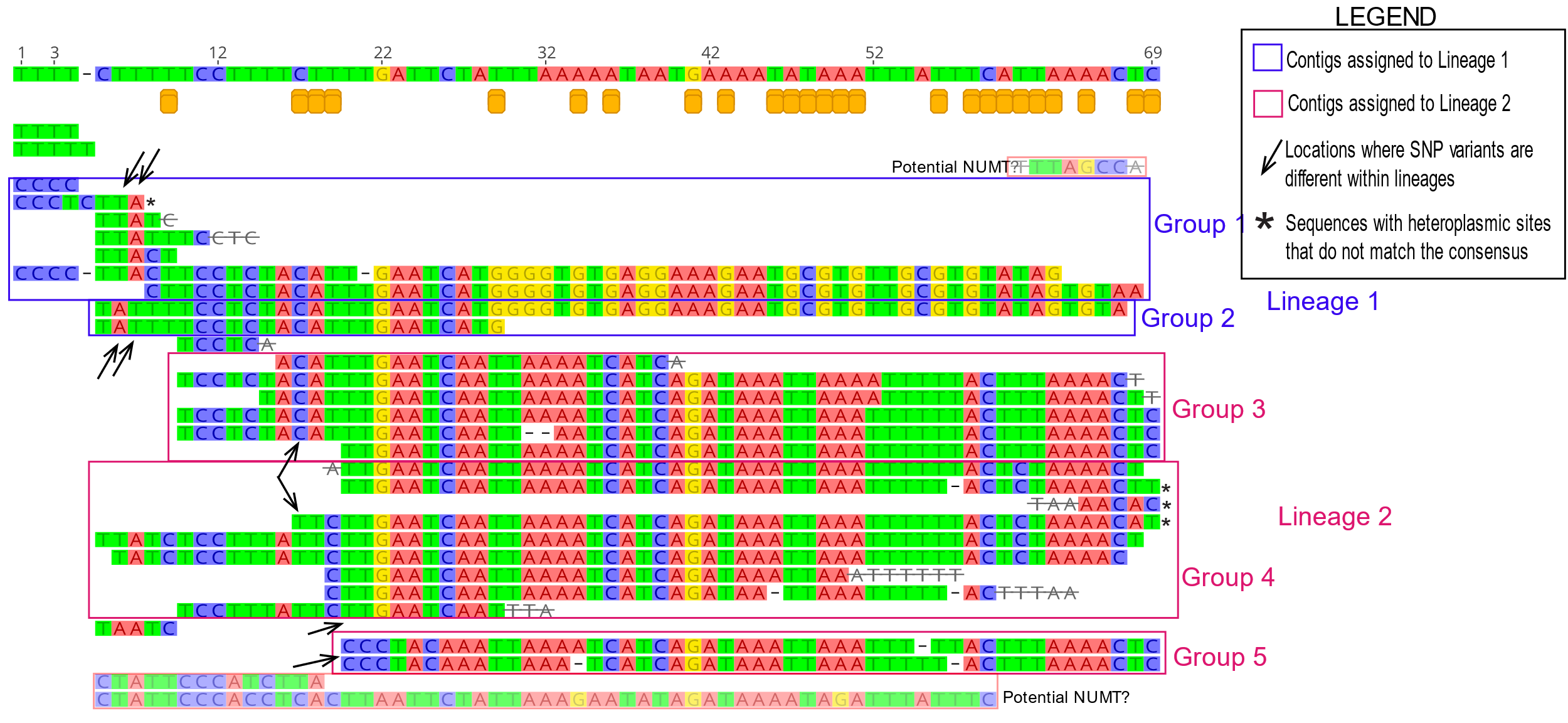


Supp. Figure 2: Ion Torrent shotgun sequence alignment of suspected heteroplasmic sites in the mitochondrial cytochrome c oxidase subunit II (COII) gene. It is important to note that the contigs presented here only consist of variable sites and have been reduced in length by ~90% of their original length (~150 – 500 bp per contig). Almost all of these variable sites are separated by long, non-variable regions shared across the dataset. These reduced-contigs have been sorted into analogues producing the two proposed heteroplasmic “lineages”. Within lineages, variable nucleotide sites have been indicated by an arrow and these contigs were classified into “groups”. Some reduced-contigs were not able to be sorted into any lineage and are suspected to be NUMTs, additional mtDNA variants, or errors.


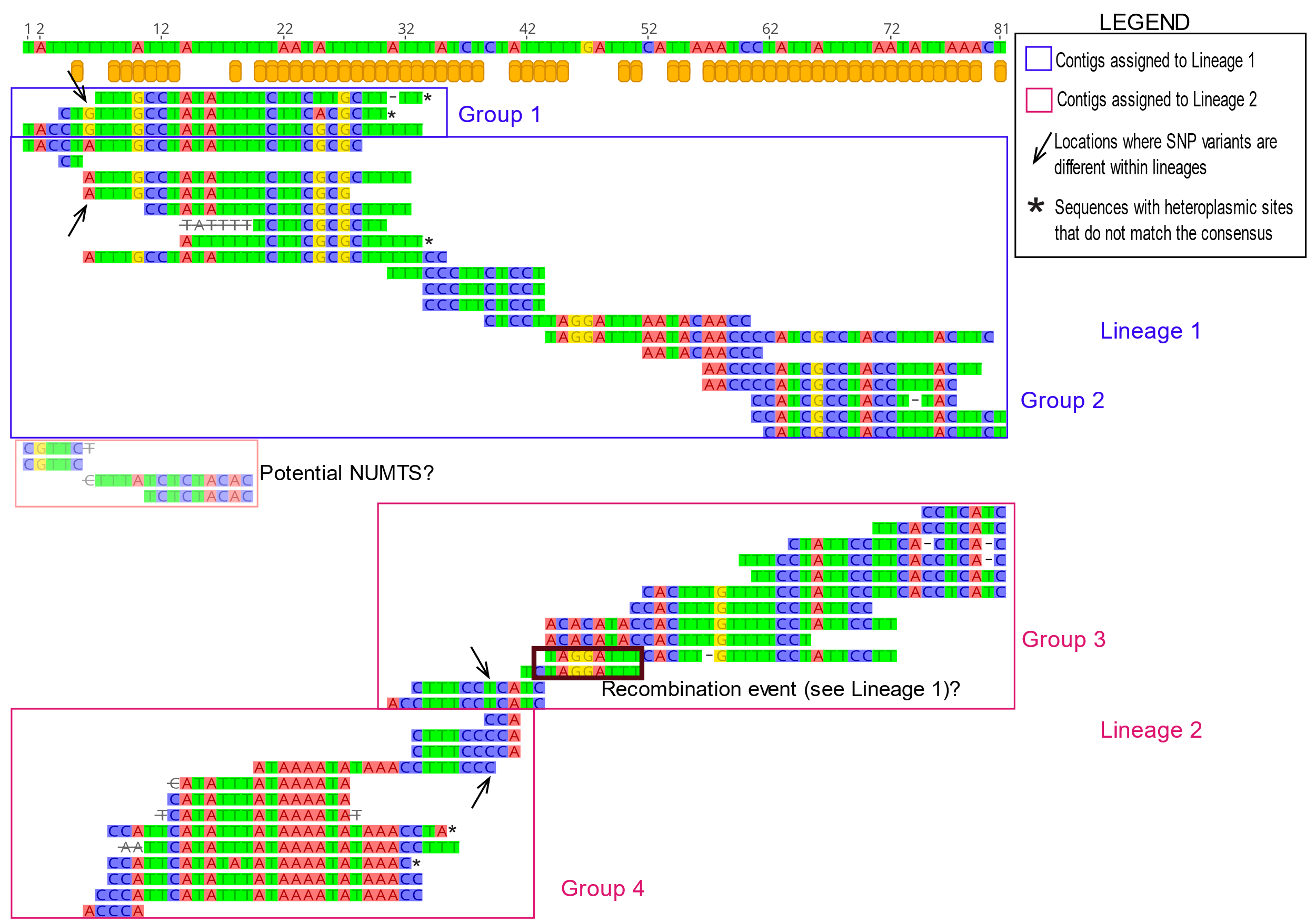


Supp. Figure 3: Ion Torrent shotgun sequence alignment of suspected heteroplasmic sites in the mitochondrial cytochrome b (cytb) gene. It is important to note that the contigs presented here only consist of variable sites and have been reduced in length by ~90% of their original length (~150 – 500 bp per contig). Almost all of these variable sites are separated by long, non-variable regions shared across the dataset. These reduced-contigs have been sorted into analogues producing the two proposed heteroplasmic “lineages”. Within lineages, variable nucleotide sites have been indicated by an arrow and these contigs were classified into “groups”. Some reduced-contigs were not able to be sorted into any lineage and are suspected to be NUMTs, additional mtDNA variants, or errors.


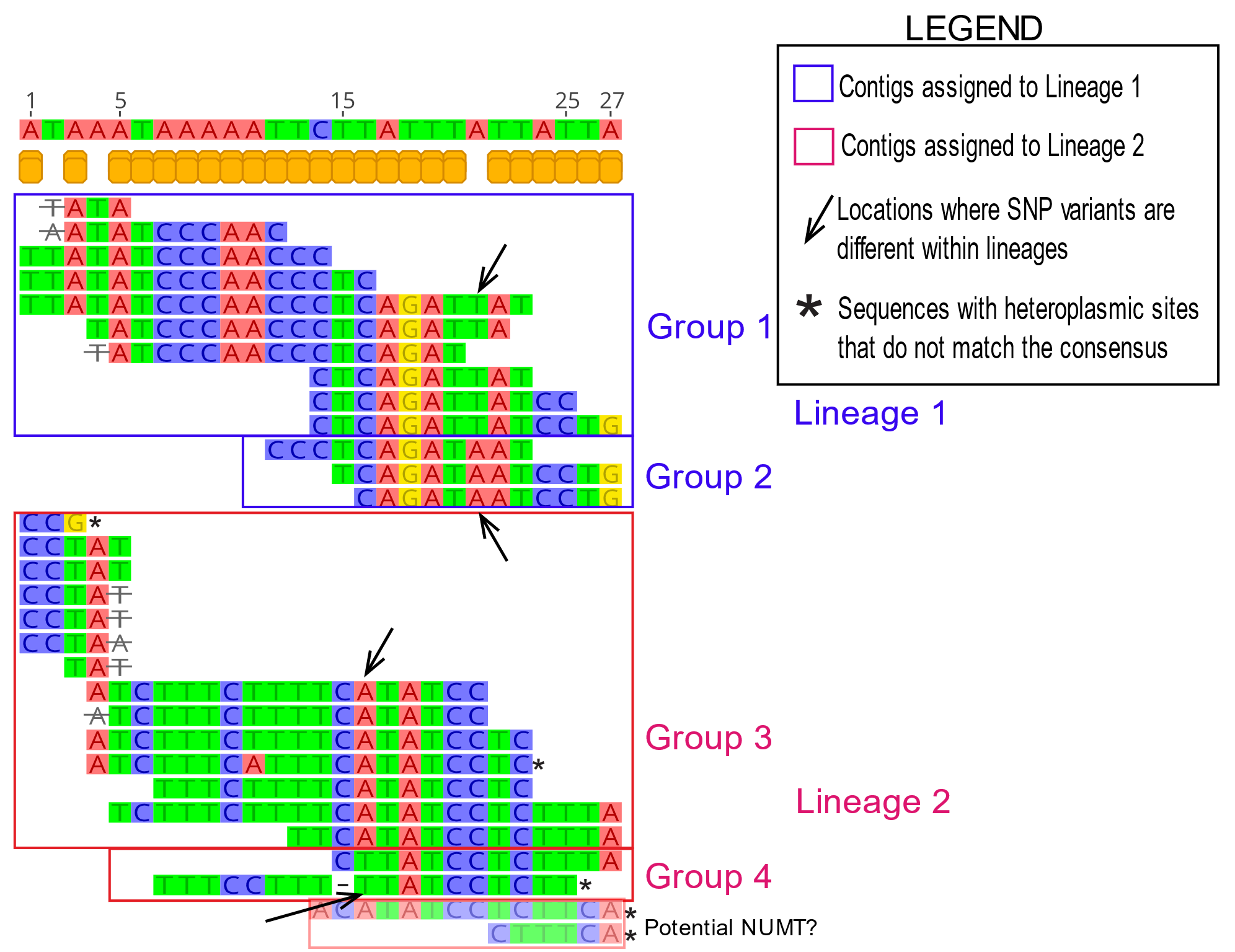


Supp. Figure 4: Ion Torrent shotgun sequence alignment of suspected heteroplasmic sites in the mitochondrial NADH-ubiquinone oxidoreductase chain 4 (ND4) gene. It is important to note that the contigs presented here only consist of variable sites and have been reduced in length by ~90% of their original length (~150 – 500 bp per contig). Almost all of these variable sites are separated by long, non-variable regions shared across the dataset. These reduced-contigs have been sorted into analogues producing the two proposed heteroplasmic “lineages”. Within lineages, variable nucleotide sites have been indicated by an arrow and these contigs were classified into “groups”. Some reduced-contigs were not able to be sorted into any lineage and are suspected to be NUMTs, additional mtDNA variants, or errors.

**Supp. Material B: Phylogenetic analysis of *Wolbachia* strains**

**B. 1. Methods: Pre-processing of phylogenetic sequences**

Both of our cloned bee specimens had two haplotypes for both non-contiguous *Wolbachia* gene regions (COI-like and *wsp*). We identified which of the two *Wolbachia* strains each of our gene fragments belonged to by comparing them with BLAST results of published *Wolbachia* genomes. One of the two haplotypes from both *Wolbachia* gene regions was highly similar to the NCBI published *Wolbachia* genomes *w*Ha and *w*CauA (>99% identify match; accession PRJNA176303 (Ellegaard et al., 2013) and PRJNA550963, respectively). We labelled the former *A. morosus*-infecting *Wolbachia* strain “*w*AmHa”. The latter sequences from each genome did not match any published (BLAST) whole *Wolbachia* genomes, and therefore we labelled this strain “*w*Amor”. We generated a concatenated 1,271 bp nucleotide alignment in Geneious version 10.2.2 with best alignment inferred from amino acids. This alignment included 15 supergroup A *Wolbachia* strains comprised of the <665 bp COI-like and <606 bp *wsp* genes; four samples from our data — two strains from both the northern- and southern-most *A. morosus* populations — and eleven trimmed *Wolbachia* genomes used by Gerth and Bleidorn (2016) (Supp. Table 4).

**B. 2. Methods: Phylogenetic analysis of *Wolbachia* strains**

To find the best partition schemes and DNA substitution models for these sequence data (nucleotide alignment of two *Wolbachia* genes; COI-like and *wsp*), PartitionFinder version 2.1.1 (Lanfear et al., 2017) was employed with an AICc (corrected Akaike information criterion) and a ‘greedy’ algorithm from the available BEAST models (Lanfear et al., 2012, Lanfear et al., 2017, Guindon et al., 2010). All codon positions for both gene fragments were assigned individual partitions. The first and third COI-like codons as well as the second *wsp* codon positions were each assigned an HKY+I+X model. The second COI-like, first and third *wsp* codon positions were assigned HKY+X, HKY+Γ+X and TRN+I+X substitution models, respectively. BEAST run files, parameters and optimisations for phylogenetic analyses were set in BEAUTi version 2.6.2 (Bouckaert et al., 2019). All partitions were assigned a relaxed log normal clock model. Known node ages were assigned based on the dated phylogeny in Gerth and Bleidorn (2016) with either a normal or log normal distribution to best fit the 95% highest posterior densities (HPDs) of each node. An additional uniform distribution was set at the boundaries of these 95% HPDs.

Phylogenetic analyses were implemented in BEAST version 2.6.2 (Bouckaert et al., 2019) with 100 million iterations, sampled every 20,000^th^ iteration. The resulting log files were analysed in Tracer version 1.7 (Rambaut et al., 2018) and a 10% burnin was employed, which was always after stationarity had been achieved. Maximum clade credibility trees were produced using TreeAnnotator version 2.6.2 (Bouckaert et al., 2019). This analysis was performed four times to check for consistent results and stationarity. All four post-burnin log and tree files were combined using LogCombiner version 2.6.2 (Bouckaert et al., 2019).

FigTree version 1.4.4 (Drummond, 2016) was used to visualize trees. Our recovered tree height was about half of that recovered in Gerth and Bleidorn (2016) (Supp. Figure 6). Gerth and Bleidorn (2016) used entire *Wolbachia* genomes and included *Wolbachia* supergroup B in their analyses, and likely recovered a more reliable tree height. To correct for this, we used FigTree to set the root height at 76 million years ago (mya) to be concordant with Gerth and Bleidorn (2016) (Supp. Figure 5).

**B. 3. Results:** The maximum-credibility tree shows high posterior probability (PP ≥ 0.95; Supp. Figure 7) values at most nodes. The uncorrected tree height was 32.7 mya (95% HPD = 26, 44.1 mya; Supp. Figure 6), but this was scaled to match the tree produced by Gerth and Bleidorn (2016) at 76 mya (95% HPD = 60.4, 102.5 mya; see methods). The Gerth and Bleidorn (2016) phylogenetic tree was dated using host divergence times and demonstrated that *Wolbachia* strains diverged within host sister species in the bee genus *Nomada* (Hymenoptera: Apidae). Our tree (Supp. Figure 5) indicates that the most recent common ancestor (MRCA) of these co-infecting *Wolbachia* strains in *A. morosus* diverged approximately 61.5 mya (95% HPD = 39.6, 85.8). The MRCA within *w*Amor strain diverged approximately 2 mya (95% HPD = 0.3, 4.1 mya) indicating a longer divergence time within this host, whereas and *w*AmHa strain diverged approximately 0.52 mya (95% HPD = 0, 1.3 mya) (Supp. Figure 5). However, the 95% HPDs overlap (Supp. Figure 5) and these results should therefore be interpreted cautiously.

Supp. Table 4: Full names and NCBI Bioproject accession numbers of all supergroup A *Wolbachia* genomes used in this study (where relevant) for phylogenetic analysis based on Gerth and Bleidorn (2016). Full genomes were trimmed into two non-contiguous gene regions (COI-like and *wsp*) and fragment lengths are given for each. Total lengths of each gene were 665 bp total and 606 bp total, respectively.

| **Name** | **Host** | **Accession** | **Reference** | **COI-like** | ***wsp*** |
| --- | --- | --- | --- | --- | --- |
| *w*AmHa | *Amphylaeus morosus* | N/A | N/A | 665 bp | 579 bp |
| *w*Amor | *Amphylaeus morosus* | N/A | N/A | 665 bp | 564 bp |
| *w*Au | *Drosophila simulans* | PRJEB6321 | Sutton et al. (2014) | 665 bp | 591 bp |
| *w*Ha | *Drosophila simulans* | PRJNA176303 | Ellegaard et al. (2013) | 665 bp | 579 bp |
| *w*Mel | *Drosophila melanogaster* | PRJNA272 | Wu et al. (2004) | 665 bp | 591 bp |
| *w*Melpop | *Drosophila melanogaster* | PRJNA196671 | Woolfit et al. (2013) | 665 bp | 591 bp |
| *w*Nfe | *Nomada ferruginata* | PRJNA322628 | Gerth and Bleidorn (2016) | 665 bp | 576 bp |
| *w*Nfla | *Nomada flava* | PRJNA322628 | Gerth and Bleidorn (2016) | 665 bp | 576 bp |
| *w*Nleu | *Nomada leucophthalma* | PRJNA322628 | Gerth and Bleidorn (2016) | 665 bp | 576 bp |
| *w*Npa | *Nomada panzeri* | PRJNA322628 | Gerth and Bleidorn (2016) | 665 bp | 576 bp |
| *w*Rec | *Drosophila recens* | PRJNA254527 | Metcalf et al. (2014) | 665 bp | 585 bp |
| *w*Ri | *Drosophila simulans* | PRJNA33273 | Klasson et al. (2009) | 665 bp | 570 bp |
| *w*Suz | *Drosophila suzukii* | PRJEB596 | Siozios et al. (2013) | 665 bp | 570 bp |


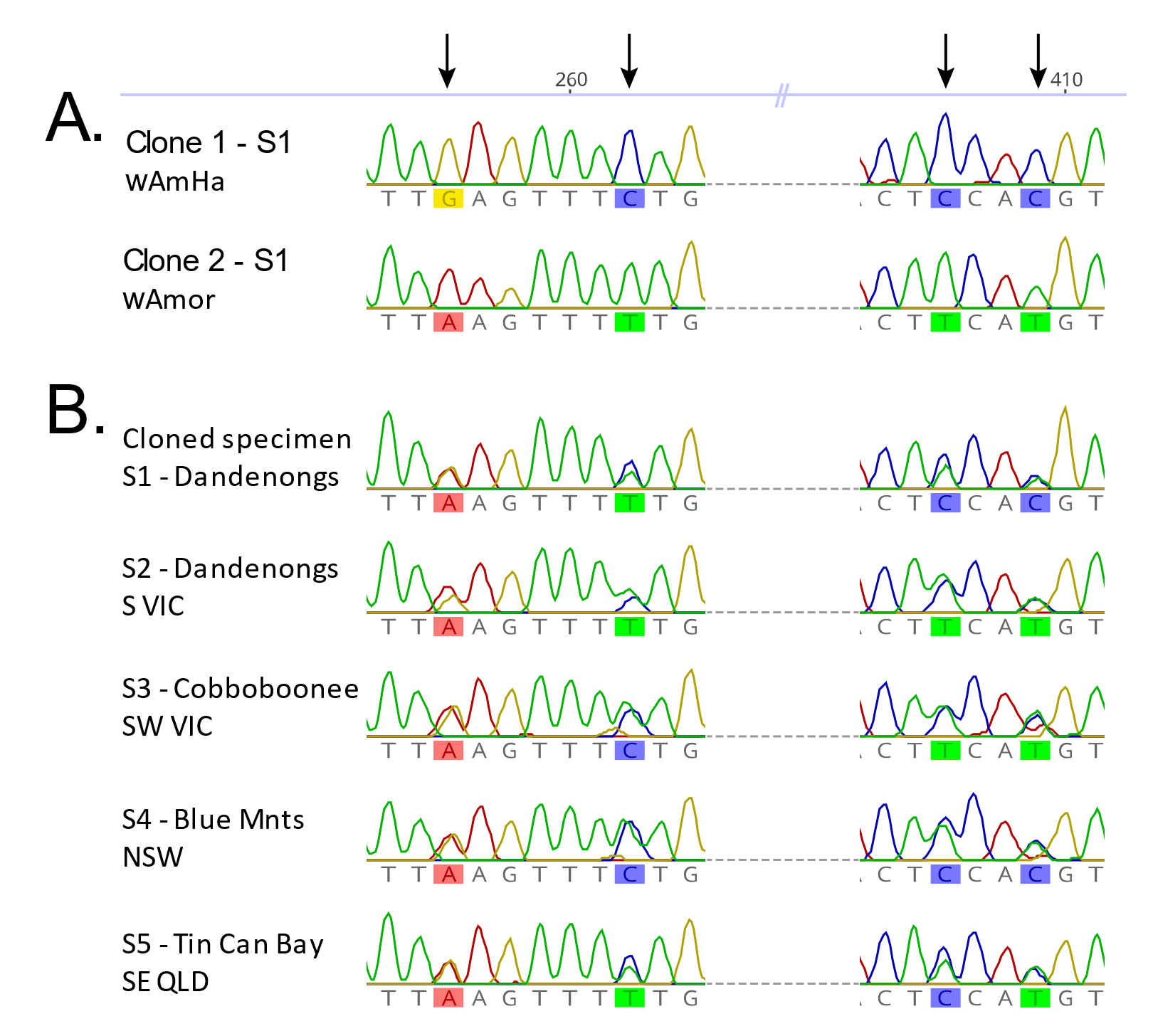


Supp. Figure 4: A. Examples of some variable nucleotide sites (indicated by arrows) in sequences recovered from a cloned individual (S1) for the *Wolbachia* COI-like gene region. B. Aligned examples of the concurrent double peaks in chromatograms from Sanger sequencing detected in a cloned specimen (S1) and four example specimens (S2–5) from four *Amphylaeus morosus* populations along the east coast of Australia. All 27 specimens sequenced had consistently occurring double peaks.

**
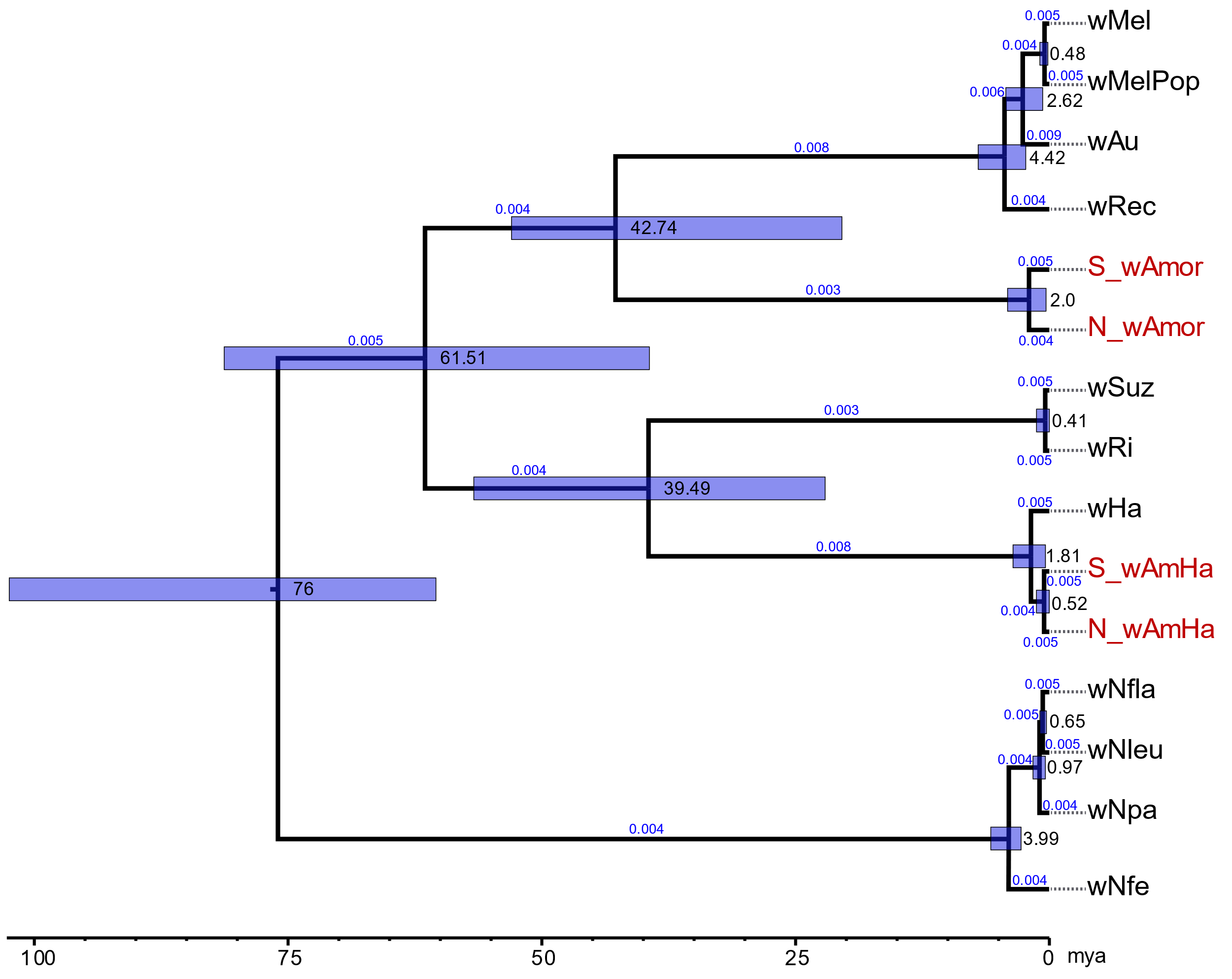
**

Supp. Figure 5: Adjusted dated phylogeny of *Wolbachia* supergroup A based on Gerth and Bleidorn (2016), with the addition of two bacterial strains (red; *w*Amor and *w*AmHa) infecting the Australian colletid bee *Amphylaeus morosus* recovered in a specimen from each the southern- (S_) and northern-most (N_) localities of its distribution. Purple bars indicate 95% highest posterior density (HPD) of node heights and values in black indicating node ages (mya). Blue text along branches indicates branch rates. Sequence data comprised two non-contiguous bacterial genes — *wsp* and COI-like (concatenated 1,271 bp).


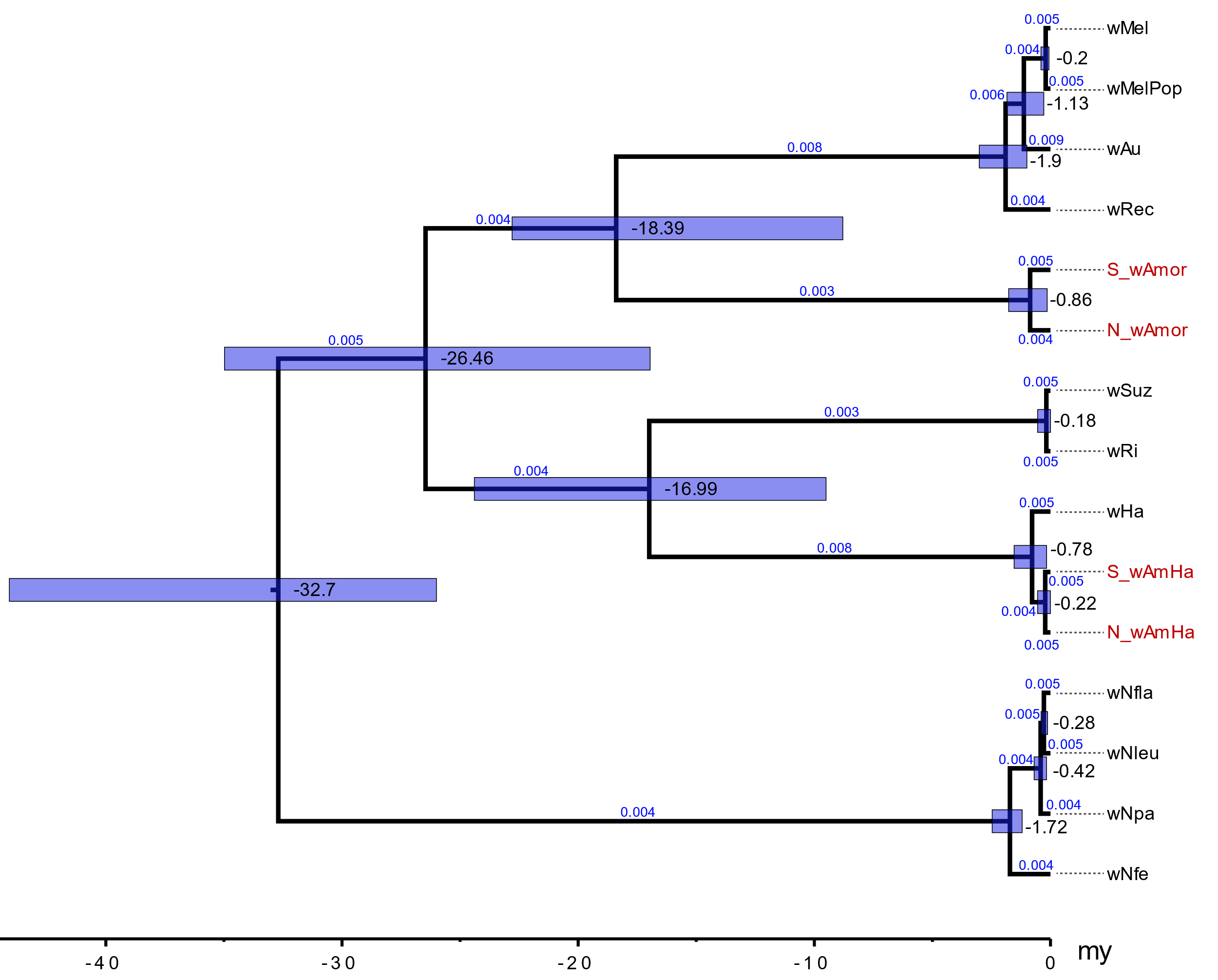


Supp. Figure 6: Dated phylogeny of Wolbachia supergroup A based on Gerth and Bleidorn (2016), with the addition of two bacterial strains (red; wAmor and wAmHa) infecting Australian colletid bee Amphylaeus morosus recovered in a specimen from each the southern- (S_) and northern-most (N_) localities of its distribution. Dates are those originally generated in our analyses, before adjustment to match those in Gerth and Bleidorn (2016). Purple bars indicate 95% highest posterior density (HPD) of node heights and black values within indicating our generated node ages (mya). Blue text along branches indicates branch rates. Sequence data comprised two non-contiguous bacterial genes — wsp and COI-like (concatenated 1,271 bp).


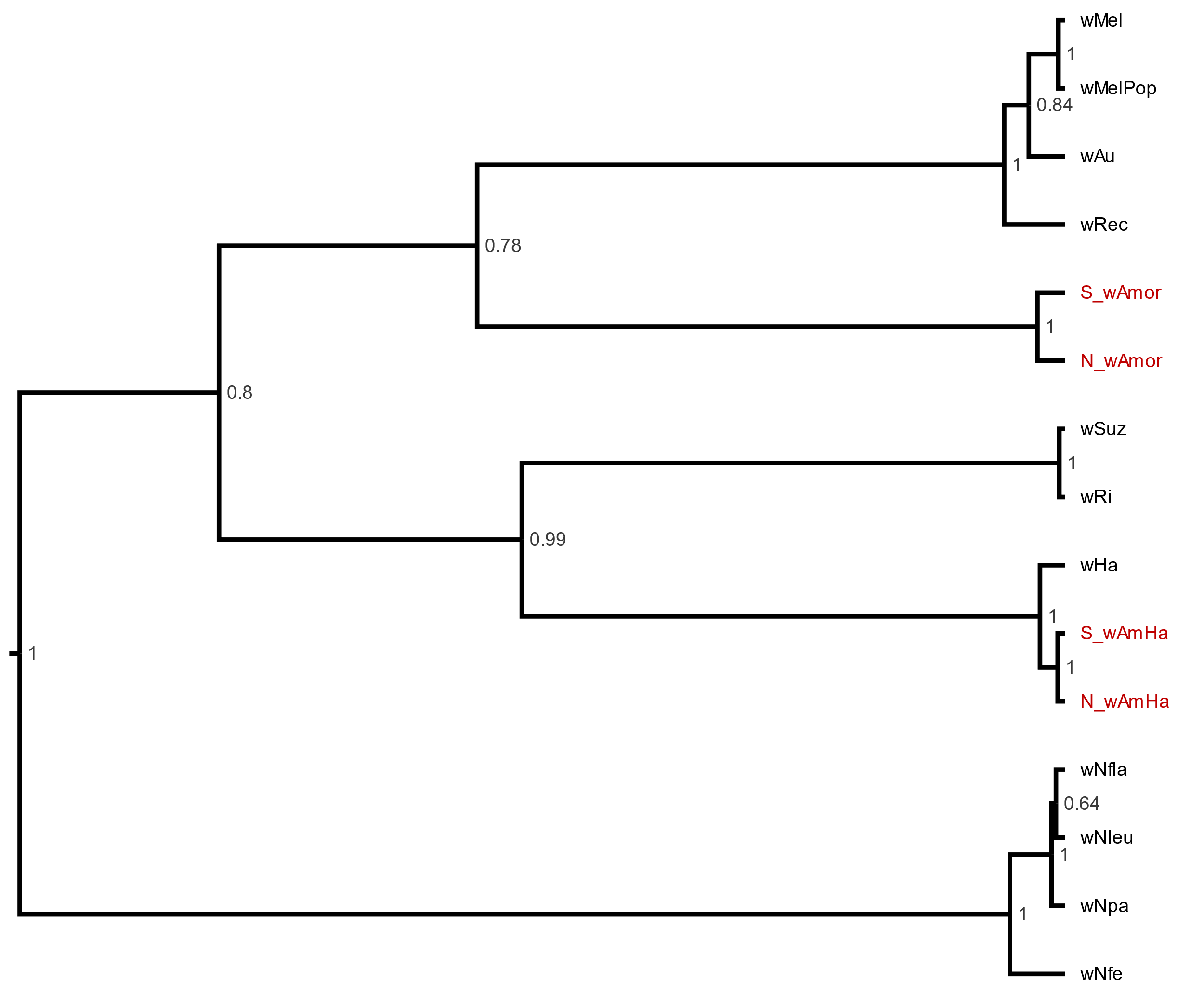


Supp. Figure 7: Phylogeny of Wolbachia supergroup A based on Gerth and Bleidorn (2016), with the addition of two bacterial strains (red; wAmor and wAmHa) infecting Australian colletid bee Amphylaeus morosus recovered in a specimen from each the southern- (S_) and northern-most (N_) localities of its distribution. Sequence data composed of two non-contiguous bacterial genes — wsp and COI-like (concatenated 1,271 bp). Posterior probabilities for each node are shown in black.

**Supplementary References**

R. Bouckaert, T.G. Vaughan, J. Barido-Sottani, S. Duchêne, M. Fourment, A. Gavryushkina, J. Heled, G. Jones, D. Kühnert, N. De Maio, M. Matschiner, F.K. Mendes, N.F. Müller, H.A. Ogilvie, L. Du Plessis, A. Popinga, A. Rambaut, D. Rasmussen, I. Siveroni, M.A. Suchard, C. Wu, D. Xie, C. Zhang, T. Stadler & A.J. Drummond, BEAST 2.5: An advanced software platform for Bayesian evolutionary analysis. PLoS Comput. Biol. 15 (2019). 10.1371/journal.pcbi.1006650.

L.M. Bragg, G. Stone, M.K. Butler, P. Hugenholtz & G.W. Tyson, Shining a light on dark sequencing: characterising errors in Ion Torrent PGM data. PLoS Comput. Biol. 9 (2013) e1003031. 10.1371/journal.pcbi.1003031.

A.J. Drummond. FigTree version 1.4.3. http://tree.bio.ed.ac.uk/software/figtree/, 2016. (accessed 25 May 2019).

K.M. Ellegaard, L. Klasson, K. Näslund, K. Bourtzis & S.G.E. Andersson, Comparative genomics of *Wolbachia* and the bacterial species concept. PLoS Genet. 9 (2013) e1003381. 10.1371/journal.pgen.1003381.

M. Gerth & C. Bleidorn, Comparative genomics provides a timeframe for *Wolbachia* evolution and exposes a recent biotin synthesis operon transfer. Nat. Microbiol. 2 (2016) 16241. 10.1038/nmicrobiol.2016.241.

S. Guindon, J.F. Dufayard, V. Lefort, M. Anisimova, W. Hordijk & O. Gascuel, New algorithms and methods to estimate maximum-likelihood phylogenies: assessing the performance of PhyML 3.0. Syst. Biol. 59 (2010) 307–21. 10.1093/sysbio/syq010.

L. Klasson, J. Westberg, P. Sapountzis, K. Näslund, Y. Lutnaes, A.C. Darby, Z. Veneti, L. Chen, H.R. Braig, R. Garrett, K. Bourtzis & S.G.E. Andersson, The mosaic genome structure of the *Wolbachia w*Ri strain infecting *Drosophila simulans*. PNAS 106 (2009) 5725–5730. 10.1073/pnas.0810753106.

R. Lanfear, B. Calcott, S.Y. Ho & S. Guindon, Partitionfinder: combined selection of partitioning schemes and substitution models for phylogenetic analyses. Mol. Biol. Evol. 29 (2012) 1695–701. 10.1093/molbev/mss020.

R. Lanfear, P.B. Frandsen, A.M. Wright, T. Senfeld & B. Calcott, PartitionFinder 2: new methods for selecting partitioned models of evolution for molecular and morphological phylogenetic analyses. Mol. Biol. Evol. 34 (2017) 772–773. 10.1093/molbev/msw260.

J.A. Metcalf, M. Jo, S.R. Bordenstein, J. Jaenike & S.R. Bordenstein, Recent genome reduction of *Wolbachia* in *Drosophila recens* targets phage WO and narrows candidates for reproductive parasitism. PeerJ 2 (2014) e529. 10.7717/peerj.529.

M.A. Quail, M. Smith, P. Coupland, T.D. Otto, S.R. Harris, T.R. Connor, A. Bertoni, H.P. Swerdlow & Y. Gu, A tale of three next generation sequencing platforms: comparison of Ion Torrent, Pacific Biosciences and Illumina MiSeq sequencers. BMC Genom. 13 (2012) 341. 10.1186/1471-2164-13-341.

A. Rambaut, A.J. Drummond, D. Xie, G. Baele & M.A. Suchard, Posterior summarization in Bayesian phylogenetics using Tracer 1.7. Syst. Biol. 67 (2018) 901–904. 10.1093/sysbio/syy032.

S. Siozios, A. Cestaro, R. Kaur, I. Pertot, O. Rota-Stabelli & G. Anfora, Draft genome sequence of the *Wolbachia* endosymbiont of *Drosophila suzukii*. Genome Announc. 1 (2013) e00032. 10.1128/genomeA.00032-13.

E.R. Sutton, S.R. Harris, J. Parkhill & S.P. Sinkins, Comparative genome analysis of *Wolbachia* strain *w*Au. BMC Genom. 15 (2014) 928. 10.1186/1471-2164-15-928.

M. Tan, R. Zhang, C. Hardman & X. Zhou, Mitochondrial genome of *Hylaeus dilatatus* (Hymenoptera: Colletidae). Mitochondrial DNA (2015) 1–2. 10.3109/19401736.2014.989511.

M. Woolfit, I. Iturbe-Ormaetxe, J.C. Brownlie, T. Walker, M. Riegler, A. Seleznev, J. Popovici, E. Rancès, B.A. Wee, J. Pavlides, M.J. Sullivan, S.A. Beatson, A. Lane, M. Sidhu, C.J. Mcmeniman, E.A. Mcgraw & S.L. O’neill, Genomic evolution of the pathogenic *Wolbachia* strain, *w*MelPop. Genome Biol. Evol. 5 (2013) 2189–2204. 10.1093/gbe/evt169.

M. Wu, L.V. Sun, J. Vamathevan, M. Riegler, R. Deboy, J.C. Brownlie, E.A. Mcgraw, W. Martin, C. Esser, N. Ahmadinejad, C. Wiegand, R. Madupu, M.J. Beanan, L.M. Brinkac, S.C. Daugherty, A.S. Durkin, J.F. Kolonay, W.C. Nelson, Y. Mohamoud, P. Lee, K. Berry, M.B. Young, T. Utterback, J. Weidman, W.C. Nierman, I.T. Paulsen, K.E. Nelson, H. Tettelin, S.L. O'neill & J.A. Eisen, Phylogenomics of the reproductive parasite *Wolbachia pipientis w*Mel: a streamlined genome overrun by mobile genetic elements. PLoS Biol. 2 (2004) e69. 10.1371/journal.pbio.0020069.
